# Epidermal Growth Factor Receptor Tyrosine Kinase Defines Critical Prognostic Genes of Stage I Lung Adenocarcinoma

**DOI:** 10.1371/journal.pone.0043923

**Published:** 2012-09-19

**Authors:** Mai Yamauchi, Rui Yamaguchi, Asuka Nakata, Takashi Kohno, Masao Nagasaki, Teppei Shimamura, Seiya Imoto, Ayumu Saito, Kazuko Ueno, Yousuke Hatanaka, Ryo Yoshida, Tomoyuki Higuchi, Masaharu Nomura, David G. Beer, Jun Yokota, Satoru Miyano, Noriko Gotoh

**Affiliations:** 1 Division of Systems Biomedical Technology, Institute of Medical Science, University of Tokyo, Minato-ku, Tokyo, Japan; 2 Human Genome Center, Institute of Medical Science, University of Tokyo, Minato-ku, Tokyo, Japan; 3 Division of Genome Biology, National Cancer Center Research Institute, Chuo-ku, Tokyo, Japan; 4 Institute of Statistical Mathematics, Tachikawa-shi, Tokyo, Japan; 5 Department of Thoracic Surgery, Tokyo Medical University, Shinjuku-ku, Tokyo, Japan; 6 Department of Surgery, Comprehensive Cancer Center, University of Michigan, Ann Arbor, Michigan, United States of America; 7 Division of Multistep Carcinogenesis, National Cancer Center Research Institute, Chuo-ku, Tokyo, Japan; University of Texas MD Anderson Cancer Center, United States of America

## Abstract

**Purpose:**

To identify stage I lung adenocarcinoma patients with a poor prognosis who will benefit from adjuvant therapy.

**Patients and Methods:**

Whole gene expression profiles were obtained at 19 time points over a 48-hour time course from human primary lung epithelial cells that were stimulated with epidermal growth factor (EGF) in the presence or absence of a clinically used EGF receptor tyrosine kinase (RTK)-specific inhibitor, gefitinib. The data were subjected to a mathematical simulation using the State Space Model (SSM). “Gefitinib-sensitive” genes, the expressional dynamics of which were altered by addition of gefitinib, were identified. A risk scoring model was constructed to classify high- or low-risk patients based on expression signatures of 139 gefitinib-sensitive genes in lung cancer using a training data set of 253 lung adenocarcinomas of North American cohort. The predictive ability of the risk scoring model was examined in independent cohorts of surgical specimens of lung cancer.

**Results:**

The risk scoring model enabled the identification of high-risk stage IA and IB cases in another North American cohort for overall survival (OS) with a hazard ratio (HR) of 7.16 (P = 0.029) and 3.26 (P = 0.0072), respectively. It also enabled the identification of high-risk stage I cases without bronchioalveolar carcinoma (BAC) histology in a Japanese cohort for OS and recurrence-free survival (RFS) with HRs of 8.79 (P = 0.001) and 3.72 (P = 0.0049), respectively.

**Conclusion:**

The set of 139 gefitinib-sensitive genes includes many genes known to be involved in biological aspects of cancer phenotypes, but not known to be involved in EGF signaling. The present result strongly re-emphasizes that EGF signaling status in cancer cells underlies an aggressive phenotype of cancer cells, which is useful for the selection of early-stage lung adenocarcinoma patients with a poor prognosis.

**Trial Registration:**

The Gene Expression Omnibus (GEO) GSE31210

## Introduction

Lung cancer is the leading cause of cancer-related death in the world. With the recent advances in diagnostic imaging technology such as computed tomography, the number of patients diagnosed with stage I non-small cell lung cancer (NSCLC), particularly adenocarcinoma, the commonest histological type, has been increasing [1], [2]. However, even among patients with the earliest form, stage IA (tumors ≤3 cm in diameter with no evidence of regional lymph node and/or regional metastasis, according to the American Joint Cancer Committee/Union Internationale Contre Le Cancer [AJCC/UICC] 6th Edition), treated by surgery with curative intent, 10–30% will relapse and die of recurrence [3]. It is also reported that 30–40% of stage I patients, including stage IA and IB, will relapse [4]. Therefore, biomarkers to identify high-risk patients with a poor prognosis among stage I patients, and who would benefit from adjuvant therapy, are greatly needed, due to the low predictive powers of clinicopathological factors to identify such patients [5].

**Figure 1:**
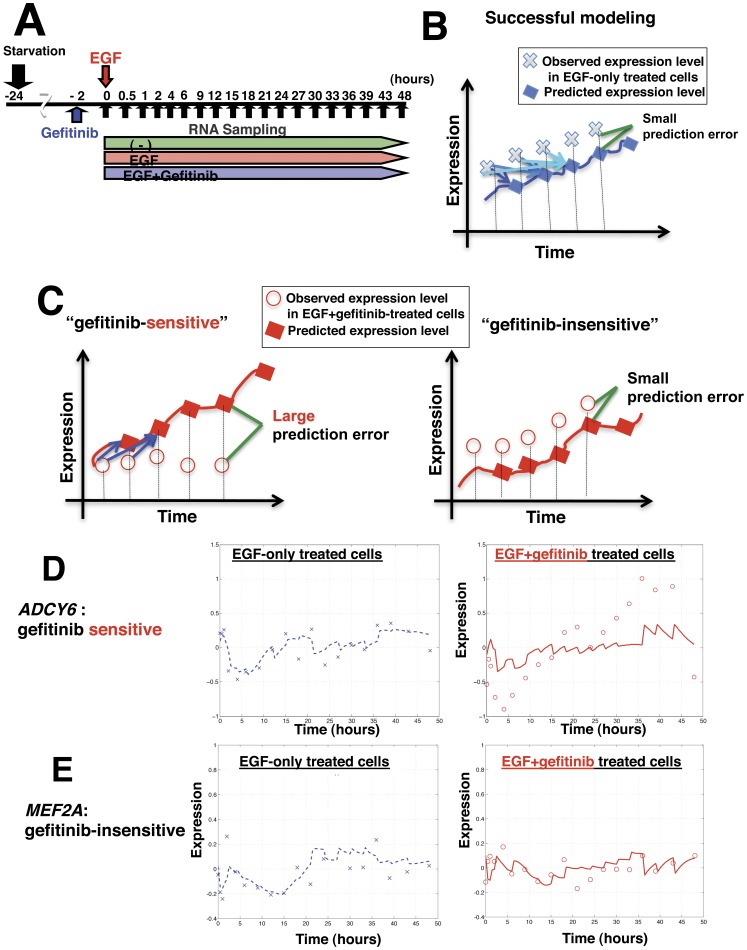
Diagrams of experimental procedures and gene set selection by SSM analysis. (A) A diagram of the *in vivo* experimental procedures. The serum-starved cells were stimulated with EGF (100 ng/mL) in the presence or absence of gefitinib (0.5 μM) for 48 h at 37°C. Before stimulation with EGF, cells were starved for 24 h at 37°C. Total RNA was isolated at each time point as indicated by the arrows (19 time points); the same experiments were performed two or three times at several time points. (B) Schematic view of time-course gene expression patterns of predicted or observed gene expression levels. The blue solid line represents a predicted gene expression pattern based on the EGF-response SSM, using the observed gene expression levels derived from the EGF-treated cells (x). (C) The red solid line represents a predicted gene expression pattern based on the EGF-response SSM, using the observed gene expression levels derived from the EGF+gefitinib-treated cells (o). (D, E) A representative gene expression pattern of gefitinib-sensitive genes (D) and -insensitive genes (E). Left panels: observed gene expression patterns in EGF-treated cells (x in blue) and EGF-response SSM-predicted gene expression patterns (blue dotted line). Right panels: observed gene expression patterns in EGF+gefitinib-treated cells (o in red) and the EGF-response SSM-predicted gene expression patterns (red solid line).

Several whole gene expression profiling studies have been conducted to obtain gene signatures applicable as biomarkers for clinical use [4], [5], [6], [7], [8], [9], [10], [11]. However, there is still little evidence to support the use of gene signatures in preference to clinical factors, including stage, age, and sex [5]. In particular, to the best of our knowledge, gene signatures that enable prediction of the outcomes of stage IA patients have not been reported.

**Figure 2:**
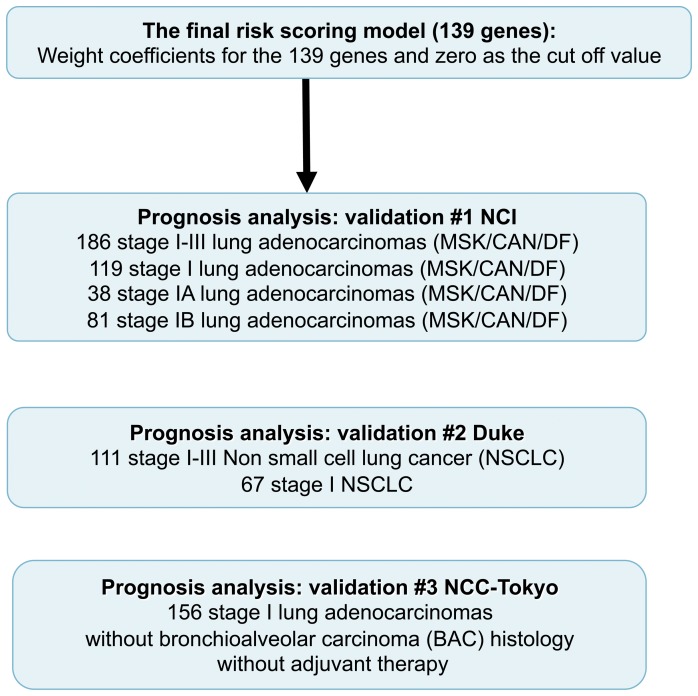
Validation procedures. Validation procedures of the final risk scoring model using the 139 genes.

Epidermal growth factor (EGF) signaling affects a variety of cellular processes linked to aggressive phenotypes of lung and other cancer cells, such as growth, invasion, and metastasis [12], [13]. EGF activates EGF receptor (EGFR) tyrosine kinase and stimulates a variety of intracellular signaling pathways. The EGF signaling pathway is considered to be commonly, but to different extents, de-regulated in lung cancer cells by oncogenic EGFR, KRAS, or BRAF mutations and/or by other unidentified genetic/epigenetic alterations. Up to now, however, such mutations/alterations themselves have not been proven useful for predicting patients' outcomes. Thus, methods to identify and assess the de-regulated EGF signaling status driven by genetic/epigenetic alterations in cancer cells are necessary. However, since it has been difficult to comprehensively identify EGF signaling-regulated genes from the huge quantity of gene expression profiling data that change dynamically over time in response to EGF [14], comprehensive assessment of the significance of EGF signaling-regulated genes in aggressive phenotypes of human cancer is lacking.

**Figure 3:**
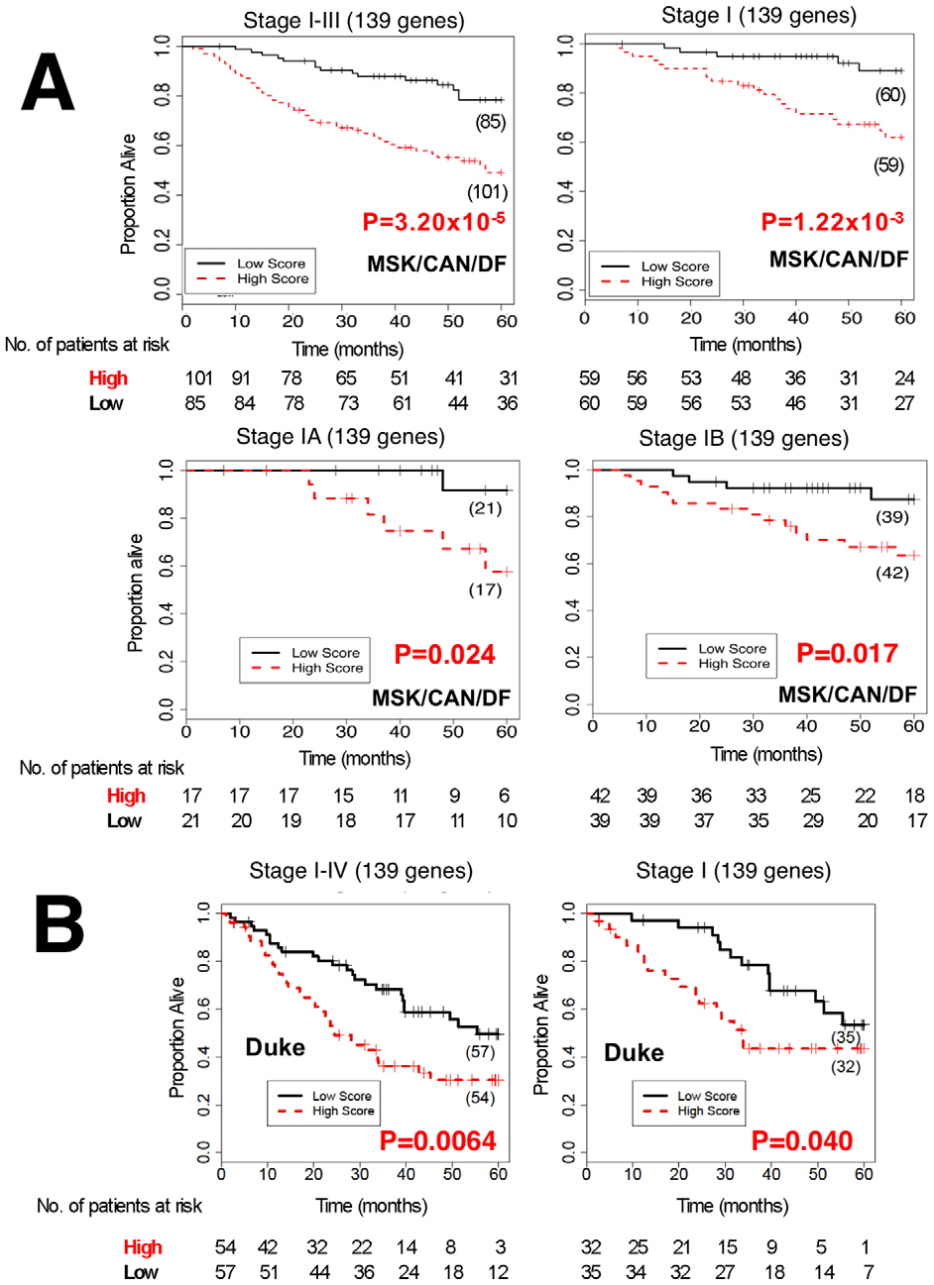
The power of survival prediction for the NCI validation data sets and the Duke data set using the 139 genes. Kaplan-Meier plot survival estimates are depicted for high- (red line) and low- (black line) risk groups by analyzing the NCI validation data sets containing two data sets designated as MSK and CAN/DF (A), and the Duke data set (B). P-values were obtained with the use of the log-rank test. Numbers in parentheses represent the number of patients that were segregated.

We used a State Space Model (SSM) to predict gene expression patterns in cells stimulated with EGF, based on a mathematical assumption that expression levels of genes in cells at one time point affect expression levels of each gene at the next time point, as we previously reported [15]. Expression levels of each gene in cells stimulated with EGF at succeeding time points are thus predictable using the observed gene expression levels at the preceding time points. When cells were stimulated with EGF in the presence of an EGFR tyrosine kinase-specific inhibitor, gefitinib [16], the expression patterns of genes that were unpredictable due to inhibition of EGFR tyrosine kinase were designated as gefitinib-sensitive genes.

**Table 1:** Gene names of the 139 genes identified for stage IA prediction and their biological functions.

Biological functions	Gene names
growth factor	*HBEGF, GDNF, PDGFB, CTGF*
cytokine	*LIF, IL1F5, TGFB2, IL1A*
chemokine and receptor	*CXCL1, CXCR7, S100A2*
angiogenesis	*VEGFA*
cytokine-related factor	*IGFBP3, IGFBP6, IGFBP2*
cell proliferation	*JUND, PHLDA2, FOSL1*
negative regulation for cell proliferation	*SESN1, INHBA, GPNMB*
cell motility	*TMSB10, CDC42EP2, LPXN, RAC2, CDC42*
cell adhesion	*ITGB8, ITGB7, CLEC2B*
apoptosis	*CASP4, NDRG1, DDX21, IER3*
cell migration	*NMU, PLA*, *ADAM10, ADAM8, CYR61, AMAM19,*
	*MMP12, TIMP3, LGALS7, MMP1, MMP3*
tyrosine kinase signaling	*PTRF, GRB10, PAK2, DUSP1, DUSP4, SPRY4, PI3KCD,*
	*DUSP5, SPRY2, SH2D2A*
actin cytoskeleton organization	*ACTC1, PALM, MYLK, MYL9, COTL1, TUBA1A*
immune response	*NFATC4, IL1RN, NFIL3, ISG20L1, ISG20*
wnt signaling	*FZD10, SOX9*
vesicular trafficking	*MLPH, HGS, VCP, FER1L4*
ubiquitination	*UBE2C, UBD*
cell cycle	*CDKN1A, SEH1L, CENPF*
signal transduction	*CSNK1D, PDIA4, YWHAQ, ITPR1*
stemness	*SOX2*
transcription	*SPDEF, ETS2, TAF9, NFIL3, SMOX, ZBED2, CD3EAP,*
	*ATF2, ZNF750, NCOR2, HOPX*
protein folding	*HSPA1A, HSPA8, HSPB1, HSPA5*
(epidermis) differentiation	*KRT14, ADRBK2, ID1, KRT5, SALL2*
G protein	*GNG4, MRAS, GNG11, GNB1*
DNA repair	*NUPR1*
tumor suppressor	*TSC22D1, MTSS1*
tumor prognostic factor	*KIAA1199*
metabolism	*MVK, BPGM, THRA, HMGA2, MTHFD2, GAPDH, UST,*
	*HMGCS1, KCNJ5, PCK2, NP, GAD1, ADCY6, SERPINB5,*
	*ODC1, PPAP2A, CHST2, SLC25A37, RDH16, PPRC1,*
	*LDLR, PFKFB3, HMGCR, ALOX15, CYP1A1, SLC29A1,*
	*SEPW1*
ribosome	*RPS14*
RNA binding protein	*HNRPM*
unknown	*DDEFL1*

The aggressiveness of lung cancer cells, defined by their abilities with respect to cell survival, invasion, and metastasis, is considered to be related to patient prognosis. In the present study, it is shown that expression signatures of such gefitinib-sensitive genes are useful to predict the outcome of early-stage lung adenocarcinoma patients. We propose that our strategy, analyzing biological pathways that involve changes in gene expression levels over time in profiles obtained by DNA microarray and next-generation sequencing, holds promise for the discovery of genes that could be used as biomarkers to predict aggressive phenotypes in cancer patients.

**Table 2:** Hazard ratios for overall survival (OS).

			Multivariate		
Data set	Case (n)	Variable	HR	95% CI	*P*
NCI (MSK/CAN/DF)					
	Stage I-III (186)	Age	1.03	1.00 – 1.06	0.028
		Sex (Male/Female)	1.54	0.96 – 2.46	0.072
		Stage (II/I)	2.45	1.43 – 4.15	0.0012
		Stage (III/I)	5.07	2.66 – 9.30	4.67E–06
		139 gene risk score (High risk/low risk)	2.91	1.75 – 5.04	2.45E–05
	Stage I (119)	Age	1.05	1.01 – 1.09	0.0062
		Sex (Male/Female)	1.06	0.51 – 2.18	0.88
		Stage (IB/IA)	1.61	0.73 – 4.06	0.25
		139 gene risk score (High risk/low risk)	3.56	1.63 – 8.60	0.0011
	Stage IA (38)	Age	1.00	0.94 – 1.07	0.91
		Sex (Male/Female)	0.31	0.02 – 1.96	0.24
		139 gene risk score (High risk/low risk)	7.16	1.20 – 136.06	0.029
	Stage IB (81)	Age	1.08	1.03 – 1.13	0.0010
		Sex (Male/Female)	1.56	0.66 – 3.76	0.31
		139 gene risk score (High risk/low risk)	3.26	1.37 – 8.63	0.0072
DUKE					
	Stage I-III (111)	Age	1.00	0.98 – 1.03	0.80
		Sex (Male/Female)	1.05	0.61 – 1.86	0.86
		Stage (II/I)	1.57	0.74 – 3.11	0.23
		Stage (III/I)	3.36	1.74 – 6.31	0.00050
		Stage (IV/I)	1.29	0.21 – 4.45	0.74
		139 gene risk score (High risk/low risk)	1.99	1.17 – 3.44	0.011
	Stage I (67)	Age	0.99	0.96 – 1.03	0.74
		Sex (Male/Female)	1.01	0.49 – 2.18	0.97
		139 gene risk score (High risk/low risk)	1.97	0.94 – 4.24	0.073
NCC-Tokyo					
	Stage I (156)	Age	1.02	0.95 – 1.11	0.59
		Sex (Male/Female)	0.71	0.25 – 2.07	0.52
		Surgery Extent (segmentectomy/lobectomy)	0.00	0.00 – .	0.69
		Tumor size (>2cm/≤2cm)	0.52	0.13 – 2.26	0.37
		Stage (IB/IA)	1.27	0.38 – 4.31	0.69
		139 gene risk score (High risk/low risk)	8.20	2.25 – 31.28	1.80E–03

HR, hazard ratio; CI, confidence interval.

## Methods

### Cell culture

Recombinant human epidermal growth factor (EGF) was purchased from Millipore (Billerica, MA, USA). Normal human small airway epithelial cells (SAECs) were purchased from Lonza Walkersville (Walkersville, MD, USA) and grown in SAGM medium (Cambrex, East Rutherford, NJ, USA).

**Figure 4:**
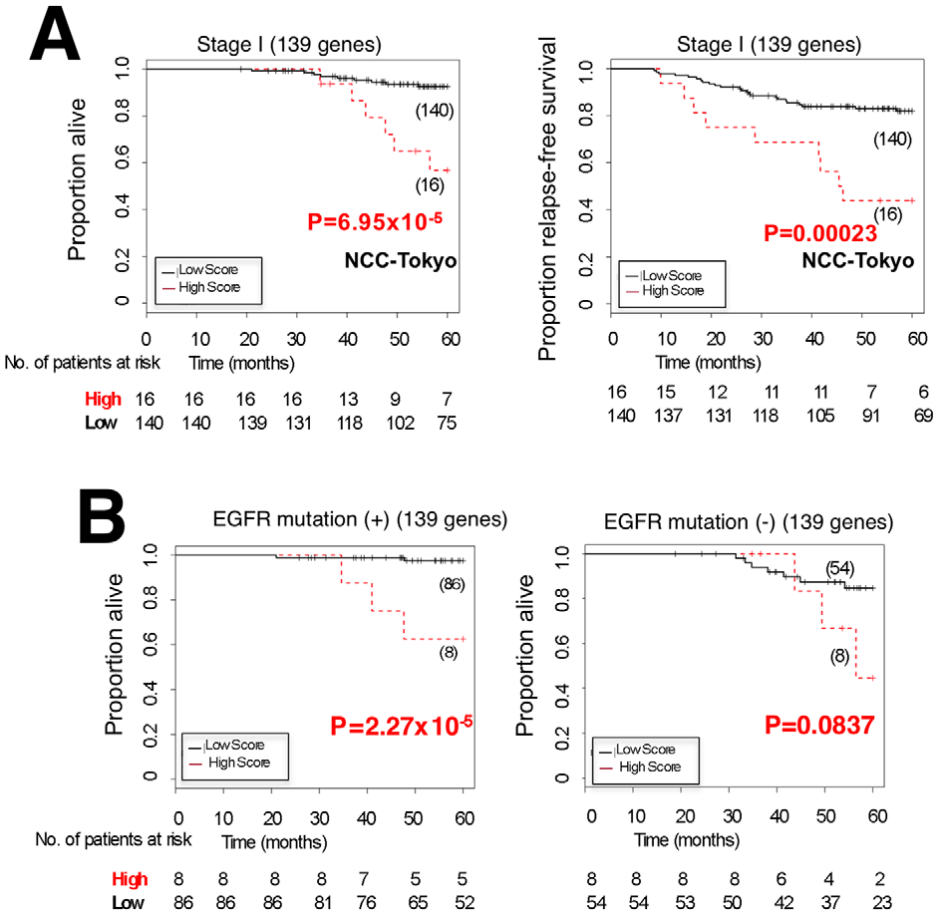
The power of survival prediction for NCC-Tokyo validation data sets using the 139 genes. (A) Kaplan-Meier plots of overall survival (OS) and recurrence-free survival (RFS) estimates for the stage I NCC-Tokyo data set without BAC histology. (B) Kaplan-Meier plot OS estimates for stage I patients in the NCC-Tokyo data set divided into EGFR mutation (+) or (−) group.

### Selection of genes with potential to influence EGF-regulated genes

A total of 1,500 genes was selected for SSM analysis as described below. First, by analyzing DNA microarray data of small airway epithelial cells (SAECs) with and without EGF treatment, 579 genes were selected based on a criterion of >1.5-fold differences in expression levels between EGF-treated and untreated cells at any time point from 0 to 9 hours, when a subset of the EGF response genes can be detected by this method [17] (Figure S1). SSM is based on a mathematical model that can predict expression patterns of each gene by using expression patterns of other genes. It represents mathematically how much each gene contributes to expression pattern of another gene. Due to the above assumption, it is not guaranteed that the expression patterns of the above 579 genes can be precisely predicted by only themselves. Thus we next conducted other approaches to further select genes that were not chosen by the criterion above, because differences in expression levels between EGF-treated and untreated cells were small. The first was a literature search (190 genes) or ingenuity pathway analysis (IPA) (802 genes), since we assume that such genes have connection with the pathways in which EGF signaling is involved; of these 190+802 genes, 597 genes whose expression at measurable levels were detected in EGF-treated cells were selected. The second approach was to select genes showing strong dynamics in expression patterns in EGF-treated cells, regardless of differences in expression levels between EGF-treated and untreated cells. A total of 324 such genes was selected from the top of the list by examining variances in expression levels in EGF-treated cells throughout the entire time course. Ultimately, 1,500 genes (579+597+324) were selected (Details are in Information S1, Selection of 1,500 genes for SSM analysis. A list of the 1,500 genes is in Table S1).

**Table 3:** Hazard ratios for relapse-free survival (RFS).

			Multivariate		
Data set	Case (n)	Variable	HR	95% CI	*P*
NCC-Tokyo					
	Stage I (156)	Age	1.01	0.96 – 1.07	0.74
		Sex (Male/Female)	0.98	0.49 – 2.03	0.96
		Surgery Extent (segmentectomy/lobectomy)	0.00	0.00 – .	0.48
		Tumor size (>2cm/≤2cm)	1.11	0.43 – 3.23	0.83
		Stage (IB/IA)	2.10	0.98 – 4.65	0.06
		139 gene risk score (High risk/low risk)	3.06	1.24 – 7.09	1.62E–02

HR, hazard ratio; CI, confidence interval.

### State Space Model (SSM) analysis

The EGF-response SSM was constructed to simulate time-course expression patterns of 1,500 genes that had the potential to be regulated by EGF (Details are in Figure S2 and Information S1, State Space Model).

**Table 4:** Potential cross-talk among pathways encoded by the 139 genes.

Signaling Category	Pathway Name*
RTK signaling	Insulin-like growth factor (IGF)-1 Signaling, VEGF Signaling
	Ephrin Receptor Signaling
Tumorigenesis	Hypoxia-inducible factor (HIF)-1α Signaling
	p53 signaling, Leukocyte Extravasation Signaling
MAPK signaling	Stress-activated protein kinase (SAPK)/Jun amino-terminal kinase
	(JNK) Signaling, ERK Signaling, p38 MAPK Signaling
Chemokine/cytokine signaling	C-X-C chemokine receptor type 4 (CXCR-4) Signaling
	Interleukin (IL)-8 Signaling,
	C-C chemokine receptor type 3 (CCR3) Signaling
	IL-17 Signaling, IL-1 Signaling, Oncostatin M Signaling
Integrin/ actin/ cytoskeleton	Integrin Signaling, Actin Cytoskeleton Signaling
signaling	Focal adhesion kinase (FAK) signaling, Tight Junction Signaling
Nuclear Receptor Signaling	Retinoic acid receptor (RAR) Activation
	Vitamin D Receptor (VDR) Activation
	Aryl Hydrocarbon Receptor Signaling
Endocytosis	Clathrin-mediated Endocytosis Signaling
G protein signaling	G Beta Gamma Signaling
Stemness	Role of NANOG in Mammalian Embryonic Stem Cell Pluripotency
	Human Embryonic Stem Cell Pluripotency
Others	Protein Kinase A Signaling, PTEN Signaling, JAK/Stat siganling
	cAMP response element binding protein (CREB) Signaling
	Phospholipase C (PLC) Signaling, Wnt/β-catenin Signaling
	Role of Nuclear factor of activated T-cells (NFAT) in Regulation of the
	Immune Response

* Representative pathways derived from the IPA analysis are listed.

For SSM analysis, a control and a case were determined, i.e., EGF-treated cells without and with gefitinib. It was assumed that, if the systems that regulate a gene differ between cells under the two conditions, the control's gene regulatory system (the SSM for the control) cannot predict the case's time-course expression pattern of the gene [15]. The SSM for the control (EGF-response SSM) was constructed using the control's time-course gene expression data. The EGF-response SSM was used to predict the control's time-course expression patterns. The integrated P-value (*P*[EGF]), which represents the overall difference between the predicted and observed expression levels at all time points, was calculated, and genes with small prediction error (*P*[EGF]>0.4) were selected as genes with successful modeling. The EGF-response SSM was then used to predict the case's time-course gene expression pattern. Of the selected genes above, genes that showed unpredictable time-course expression patterns using the EGF-response SSM were further selected as gefitinib-sensitive genes. Such genes had integrated P-values (*P*[EGF+gefitinib])<0.05, showing a large difference between the predicted and observed gene expression patterns. In addition, genes with small prediction error (*P*[EGF+gefitinib]>0.5) were selected as gefitinib-insensitive genes. Further details are in Information S1, SSM analysis.

### Construction of the risk scoring model based on gene signature and validation of the risk scoring model

The risk scoring model to calculate a risk score for each patient was constructed using a publicly available data set (the combined data of 253 cases from University of Michigan [UM] and Moffitt Cancer Center [HSM] in the National Cancer Institute [NCI] training set) as a training data set, as previously reported [18]. A partial Cox regression method was used to decrease the complexity of the model by a partial least squares regression to avoid overfitting when more genes than the number of samples [19] are used, as previously reported [18]. The risk score is the sum of the expression scores, which are the product of the weight coefficient and expression level of each gene in the cancer tissue. Weight coefficients were determined so that the patients were classified as accurately as possible into two groups based on whether the risk score was >0 (worse prognosis) or ≤0 (better prognosis), using zero as the cut-off value. For validation, risk scores were calculated for each patient in the validation data sets using the expression level of each gene in the cancer tissue, according to the risk scoring model described above. Each patient was classified into a>0 or ≤0 risk score group using the weight coefficients and zero as the cut-off value, both of which were fully specified by the NCI training data set. (Information S1, Survival analysis in detail).

This study was conducted according to the principles of the Declaration of Helsinki, and it was approved by the institutional review boards of the National Cancer Center and the Institute of Medical Science, University of Tokyo.

## Results

### Construction of EGF-response SSM in cells stimulated with EGF

To identify EGF-regulated genes in general, human primary small airway epithelial cells (SAECs), which are cultured normal lung epithelial cells (Figure S3 and Information S1, Results, Profiles of human primary SAECs), were used. SAECs were stimulated with EGF in the presence or absence of gefitinib, and RNAs were extracted at 19 time points in a 48-hour time course, corresponding to 2 rounds of cell cycles (Fig. 1A). Gene expression profiling was then performed by DNA microarray. Genes that have the potential to be regulated by EGF were then selected for SSM analysis. Since the number of genes needs to be reduced for efficient computation, 1,500 candidate genes were selected, as described above.

The “EGF-response SSM” was then constructed using the expression levels of the 1,500 individual genes in EGF-treated cells throughout the entire time course. It was assumed that expression levels of all 1,500 genes involved in EGF signaling at one time point affect expression levels of each gene at the next time point [15]. Expression levels of each gene among the 1,500 genes in cells stimulated with EGF at succeeding time points are thus predictable using the observed expression levels of the 1,500 genes at the preceding time points. In other words, the EGF-response SSM allowed prediction of expression patterns of the 1,500 genes in EGF-treated cells.

### Selection of gefitinib-sensitive genes by SSM analysis

Of the 1,500 genes, successfully modeled genes, those for which there was little difference between predicted and observed expression patterns in EGF-treated cells, were selected first. From the successfully modeled genes in EGF-treated cells, genes for which expression patterns were not well predicted in EGF+gefitinib-treated cells were then selected. The selected genes were then designated as gefitinib-sensitive genes.

At first, expression levels of each gene at any time point were predicted based on the observed expression levels of the 1,500 genes at the preceding time points in the EGF-treated cells using the EGF-response SSM (Fig. 1B, Blue arrows indicate the observed points used for calculation [tail] and the predicted points [head], respectively). More than half of the 1,500 genes showed small prediction error and were selected as successfully modeled genes in EGF-treated cells (Fig. 1B). Gefitinib-sensitive genes were then identified from this subset. Expression levels of each gene at succeeding time points were predicted based on the observed expression levels in EGF+gefitinib-treated cells at the preceding time points using the EGF-response SSM (Fig. 1C, left panel; Blue arrows indicate the observed points used for calculation (tail) and the predicted points (head), respectively). Using this strategy, 277 genes were selected as gefitinib-sensitive genes (Fig. 1C, left panel). These genes showed large differences (large prediction errors) between the predicted and observed expression patterns. As a control, 431 genes were selected as gefitinib-insensitive genes (Fig. 1C, right panel).

Time-dependent expression patterns of either gefitinib-sensitive or gefitinib -insensitive genes are depicted in Figure 1D and 1E. The EGF-response SSM correctly predicted expression patterns of both *ADCY6* and *MEF2A* genes in EGF-treated cells and that of *MEF2A* in EGF+gefitinib-treated cells, but not that of *ADCY6* in EGF+gefitinib-treated cells.

### Risk scoring model based on gefitinib-sensitive genes as predictors of the prognosis of lung adenocarcinoma cases

The gene expression profiles of a total of 439 lung adenocarcinoma tissues derived from the NCI consortium project were used [18]. These data provided independent training and validation data sets derived from lung adenocarcinoma samples collected and analyzed in 5 different institutions and hospitals (Clinicopathological characteristics are shown in Table S2).

A risk scoring model for overall survival (OS) was constructed based on the 277 gefitinib-sensitive genes using the training data set of 253 stage I-III lung adenocarcinomas, as previously done [18] (Training set in Table S2). The partial Cox regression method was used to avoid overfitting when handling more genes than the number of samples [19]. A weight coefficient, representing the contribution level to prognosis, was determined for each gene using the NCI training data set. A larger number means a stronger effect, and positive and negative numbers mean bad and good effects on prognosis, respectively. Then, we simply removed genes with small absolute values of the weight coefficients from the 277 genes, since they are not evidently contributing to prognosis. For the above selection, we set a single threshold value (5.0E-5) for the absolute values of the weight coefficients of the 277 genes. We simply removed 138 genes with small absolute values of the weight coeffients (<,5.0E-5). As a result, we removed a half of the genes (138 genes) and 139 genes are remained. A risk scoring model was then reconstructed based on the remaining 139 genes as the final prognostic model using the same training data set (the 139 genes are in Table 1). (The weight coefficients of the 139 genes and the gene names of the 277 and 431 genes are in Table S1 saved in an Excel file. Risk scores in the training data set are in Table S3). The final prognostic model based on the 139 genes was tested for effectiveness on the independent validation data sets using the weight coefficients and zero as the cut-off value, both of which were determined by the training data set as described above (Fig. 2) First, the 186 remaining subjects from the NCI consortium project, i.e., a combined data set of Memorial Sloan-Kettering Cancer Center [MSK] and Dana-Faber Cancer Institute [CAN/DF] data sets (Clinicopathological characteristics of NCI sets are shown in Table S2) were used. The model based on the 139 genes correctly classified the stage I-III lung adenocarcinoma cases based on the log-rank test (Fig. 3A). In the Cox proportional hazard model, high-risk group cases showed a significantly shorter OS than did low-risk group cases (HR, 2.91; 95% confidence interval [CI], 1.75–5.04), P = 2.5×10^−5^) (Table 2). The model also enabled prediction of the prognosis of stage I, IA, and IB cases (Fig. 3A and Table 2). Next, the cases were divided into two independent data sets, MSK and CAN/DF, representing different institutions where samples were collected, as analyzed in the previous report [18]. The model enabled prediction of stage I-III, as well as stage I, patients (Figure S4A, S4B). The area under the curve (AUC) for each group also revealed the predictive value of the risk scoring model based on the 139 genes (MSK, stage IA AUC = 0.829, CAN/DF, stage IA AUC = 0.944; MSK, stage IB AUC = 0.813, CAN/DF, stage IB AUC = 0.574). (The risk scores in the MSK test set are in Table S4 and those in the CAN/DF test set are in Table S5).

The final risk scoring model was also validated using the publicly available Duke cohort [20]. The publicly available Duke cohort is comprised of 111 NSCLC cases, including 67 stage I cases (Clinicopathological characteristics are shown in Table S2). The risk scoring model based on the 139 genes enabled prediction of the prognosis of stage I-III, as well as stage I NSCLC, cases (Fig. 3B). In the Cox proportional hazard model, high-risk group cases showed a significantly or marginally significantly shorter OS than low-risk group cases in stage I-III and stage I cases, respectively (Table 2). Due to the limited number of cases and the lack of stage IA/IB information in a subset cases, predictive values for stage IA and IB cases were not investigated.

### Validation of the final risk scoring model based on 139 genes as predictor of prognosis of stage I cases excluding BAC histology in the NCC-Tokyo cohort

A limitation of the NCI data sets is that they include a significant number of patients (34 patients) treated with adjuvant therapy, which may modify prognosis [5]. The information about adjuvant therapy is not available for the Duke data sets. It is reported that subtypes of lung adenocarcinoma with BAC histology have a better prognosis than other subtypes. Therefore, another data set of the NCC-Tokyo cohort in which no patients underwent adjuvant therapy and information of BAC histology is available for every patient [21] was used. Information for recurrence-free survival (RFS) is also available for the data set. All 156 stage I lung adenocarcinoma cases were analyzed after excluding 6 cases with BAC histology in the data set (Clinicopathological characteristics are shown in Table S2, and details of patient selection are described in Figure S5 and Information S1, NCC-Tokyo cohort). The risk scoring model that was prospectively determined based on the 139 genes identified was applied to the cohort set. The log-rank and Cox proportional hazard tests revealed that the model enabled prediction of prognosis of OS and RFS stage I lung adenocarcinoma cases without BAC histology (Fig. 4A, Tables 2 and 3). (The risk scores in the NCC-Tokyo test set are in Table S6.

### Validation of the risk scoring model based on 139 genes in a separated group of the NCC-Tokyo cohort including 162 stage I cases with or without *EGFR* mutation

Given that the 139 genes are gefitinib-sensitive genes, it would be interesting to examine whether the risk scoring model based on the 139 genes classifies the levels of aggressiveness of tumors that are highly dependent on EGF signaling. Since the majority of tumors with activating *EGFR* gene mutations is sensitive to gefitinib, these tumors are highly dependent on EGF signaling [22]. Since all of the 156 stage I NCC-Tokyo cohort patients were informative for *EGFR* mutation status, survival analysis was performed after dividing stage I samples according to *EGFR* mutations. The model enabled prediction of the outcomes both of patients with and without *EGFR* mutations significantly or with a marginal significance (Fig. 4B). Therefore, the 139 genes can predict the aggressiveness of lung adenocarcinomas irrespective of *EGFR* mutations.

## Discussion

In the present study, to the best of our knowledge, SSM was used for the first time to analyze growth factor signaling. This strategy allowed the identification of critical prognostic genes for stage I lung adenocarcinoma patients.

Many molecules encoded by the 139 genes, such as vascular endothelial growth factor A (VEGFA), a known molecular target [23] (Table 1), are known to play roles in tumor aggressiveness. IPA analysis showed that molecules encoded by the 139 genes play roles in multiple signaling pathways that may affect properties of cancer cells: RTK signaling, chemokine/cytokine signaling, integrin/actin cytoskeleton signaling, G-protein signaling, stemness, and so forth (Table 4, Table S7. Methods are described in Information S1, IPA to identify overlapping pathways with the 139 genes). The molecules encoded by the 139 genes were compared with the 5-gene signature that consisted of *DUSP6, MMD, STAT1, ERBB3,* and *LCK* [11]. The 139 molecules include DUSP1, DUSP4, and DUSP5, the same family of proteins with DUSP6, LIF, an activator of STAT1, and HBEGF, a ligand for ERBB3 heterodimer. This indicates that at least 3 molecules in the 5-gene signature are involved in similar signaling pathways in which the 139 molecules are involved, though it is not likely that the 5-gene signature is able to predict the prognosis of stage IA cases. It would be possible to reduce the number of genes by selecting the most significant genes to develop a quantitative real time-polymerase chain reaction (PCR)-based diagnostic kit.

Because exact gene expression patterns were examined in EGF-treated cells over multiple points for 48 h, a relatively long time course, it was possible to detect genes that had altered expression levels by gefitinib-treatment not only at early time points but also at later time points. An example is shown in Figure 1D (compare observed expression levels in EGF-treated cells [left] and in EGF+gefitinib-treated cells [right]). Moreover, this method allowed identification of some gefitinib-sensitive genes, irrespective of different levels in observed gene expression between EGF-treated cells and EGF+gefitinib-treated cells, if the predicted expression patterns of the genes in the EGF response SSM are under the strong influence of other genes that showed altered expression levels in EGF+gefitinib-treated cells.

It is thus reasonable to speculate that we were able to detect indirectly regulated genes through activation of other signaling pathways after the first wave of direct activation of EGF signaling pathways. This seems to be important for identifying key genes that affect tumor aggressiveness in general. Indeed, the risk scoring model based on the 139 genes enabled identification of high- and low-risk groups irrespective of gefitinib-sensitive *EGFR* mutation status in lung adenocarcinoma tissues. The fact that the model enabled prognosis prediction even without the *EGFR* mutation further confirms that the 139 genes reflect tumor aggressiveness in general.

Each of the 139 genes has a specific weight coefficient that represents contribution levels for prognosis. Seventy genes, half of the 139 genes, have positive weight coefficients, and 69 genes, the other half of the 139 genes, have negative weight coefficients (Table S1). This indicates that our signature represents either a worse or better prognosis. It is well known that EGF signaling not only activates pathways for positively regulating tumorigenesis, but it also activates many negative regulators [17]. The levels of imbalance of activation status between the positive and negative pathways might represent cancer status, in other words, prognosis.

In contrast, when the prognostic ability of the 431 gefitinib-insensitive genes was examined, they did not have prognostic value (Information S1, Results, Comparison of the risk scoring model based on the gefitinib-sensitive genes and gefitinib-insensitive genes, Figure S6). As a comparison, the prognostic value of several sets of genes that were conventionally selected based on more than 2-fold differences in expression levels between EGF- and EGF+gefitinib-treated cells for 6 or 12 hours was also examined. However, all gene sets failed to predict prognosis in stage I patients from the MSK and CAN/DF data sets (data not shown).

Recently, guidelines have been proposed to address problems in studies seeking to develop prognostic gene signatures [5]. The present study complied with these guidelines, including use of validation and statistically significant improvement over standard risk factors (clinical variables). In addition, the number of genes used for risk score construction, 139, is small enough for a diagnostic kit, as evidenced by the 70-gene signature kit that is clinically used for breast cancer prognosis. It would be also possible to reduce the number of genes to develop a quantitative real time-polymerase chain reaction (PCR)-based diagnostic kit. It is also necessary to re-construct a risk scoring model by optimizing for the qRT-PCR test to predict RFS. We probably need to use a training set that includes full data of recurrence. We continue to collect more samples for analysis.

## Supporting Information

Figure S1 **Gene selection procedure.**
(JPG)

Figure S2 **The eight module pairs in the EGF-signaling SSM.** The time course changes in the expression levels of the 1,500 genes are classified into 8 expression patterns, called modules, that include a group of genes showing similar expression patterns. The most representative 100 genes for each module are shown. The expression pattern of each gene is vertically arranged. Each module is composed of two pairs of sub-modules that contain mirrored images of time-course gene expression patterns. Green indicates low expression compared to the average expression of each gene, and red indicates high expression compared to the average. Based on the assumption that genes belonging to the same module are under similar regulatory mechanisms, genes in a module regulate genes in every other module at each time point by the estimated regulation coefficients that are defined for each module (the estimated regulation coefficients are indicated as numbers on the red and blue arrows indicating positive and negative regulations, respectively).
(JPG)

Figure S3 **Profiles of human primary small airway epithelial cells (SAECs).** (A) Expression levels of the epidermal growth factor receptor (EGFR) family members. Western blotting was performed using specific antibodies, as indicated on the right. (B) Phosphorylation of EGFR, Akt, and ERK, upon stimulation with EGF, in various lung cancer cell lines and SAEC. After starvation for 24 h at 37°C, the cells were stimulated with EGF (100 ng/mL) for 5 min at 37°C. Western blotting was performed using specific antibodies, as indicated on the right. “P” indicates “phosphorylated.” (C) Cell growth inhibition by gefitinib in a dose-dependent manner. Cell numbers were determined using a CellTiter 96® after incubation at 37°C for 72 h with a growth medium containing gefitinib. The results represent the means ± S.D. of several independent experiments.
(JPG)

Figure S4 **Kaplan-Meier plot survival estimates.**
(JPG)

Figure S5 **Selection of eligible cases of the NCC-Tokyo cohort consisting of 156 stage I lung adenocarcinoma patients without BAC histology and adjuvant therapy (22).**
(JPG)

Figure S6 **Comparison of the risk scoring model based on the gefitinib-sensitive genes and gefitinib-insensitive genes.** A risk scoring model based on the 431 geftinib-insensitive gene signature was constructed. The prognostic ability of the two models: the 277 gefitinib-sensitive gene signature and 431 gefitinib-insensitive gene signature by using the two validation test sets are presented. The 277-gene signature was useful for predicting the survival of patients at all stages in both validation data sets and for the stage I MSK data set (P<0.05, as indicated in red in Figure S6A–C), except for the stage I CAN/DF data set (P = 0.081 in Figure S6D). Conversely, it was not possible to predict the survival for any stage at all when the 431 gefitinib-insensitive gene signature was used (high P-values, P>0.1 in Figure S6E–H).
(JPG)

Table S1 **1,500 genes with integrated P-values calculated by using the SSM and the probe ID on the Affymetrix U133A.**
(XLS)

Table S2 **Clinicopathological characteristics of non-small cell lung cancer (NSCLC) subjected to prognosis analysis.**
(XLS)

Table S3 **Risk scores in the training data set for the 139 genes for survival prediction.**
(XLS)

Table S4 **Risk scores in the MSK test set of stage I disease for the 139 genes for survival prediction.**
(XLS)

Table S5 **Risk scores in the CAN/DF test set of stage I disease for the 139 genes for survival prediction.**
(XLS)

Table S6 **Risk scores in National Cancer Center Hospital test set of stage I disease for the 139 genes for survival prediction.**
(TXT)

Table S7 **Ingenuity pathway analysis to identify overlapping pathways with the 139 genes.**
(XLS)

Information S1 **Supporting information.**
(DOC)
