# Supplementary figures and images for: Epidermal Growth Factor Receptor Tyrosine Kinase Defines Critical Prognostic Genes of Stage I Lung Adenocarcinoma
Source: PLoS One. 2012 Sep 19;7(9):e43923. doi: 10.1371/journal.pone.0043923 (PMC3446964; doi:10.1371/journal.pone.0043923)

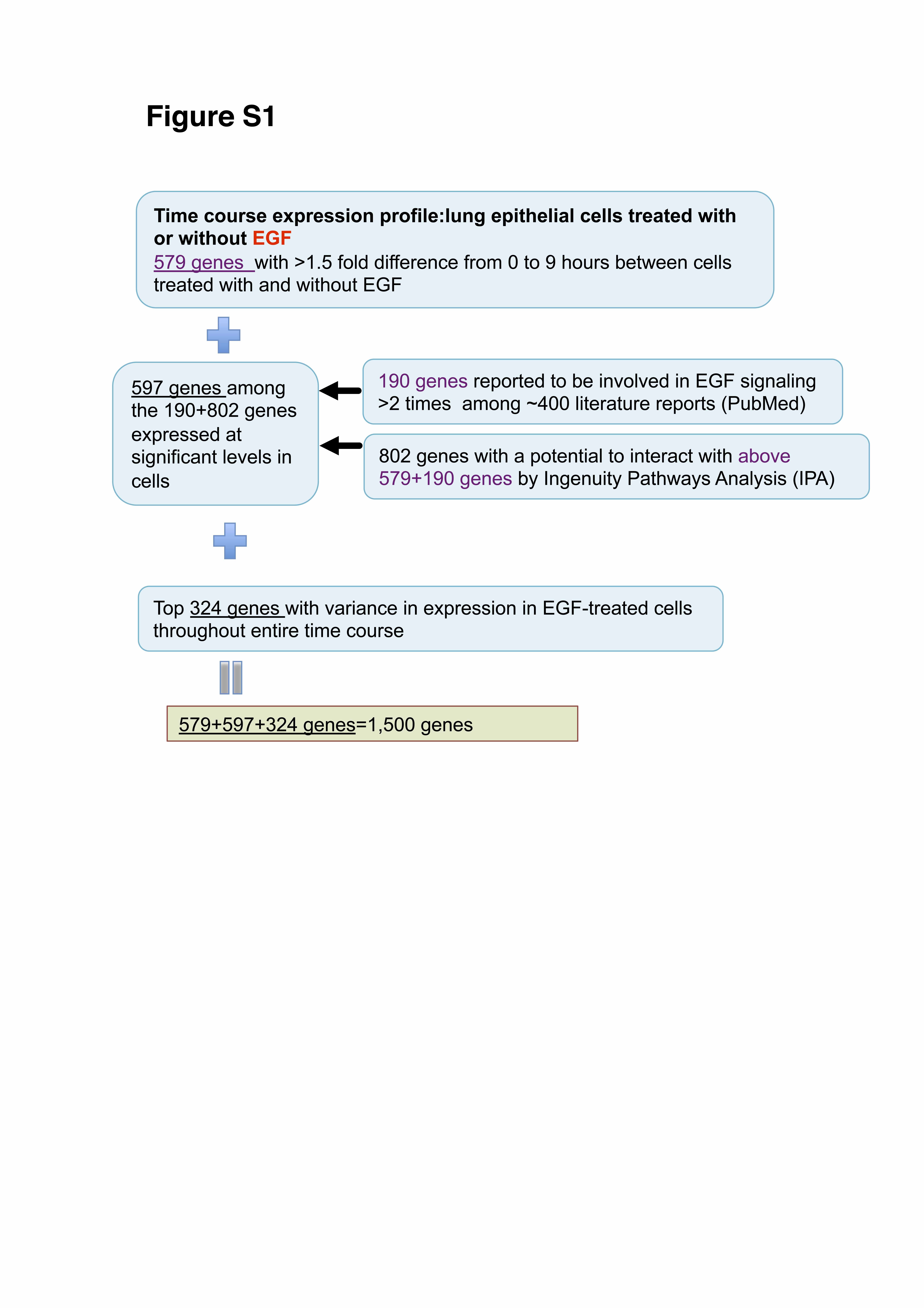

Supplement: Figure S1 — Gene selection procedure. (JPG) [file pone.0043923.s001.jpg]

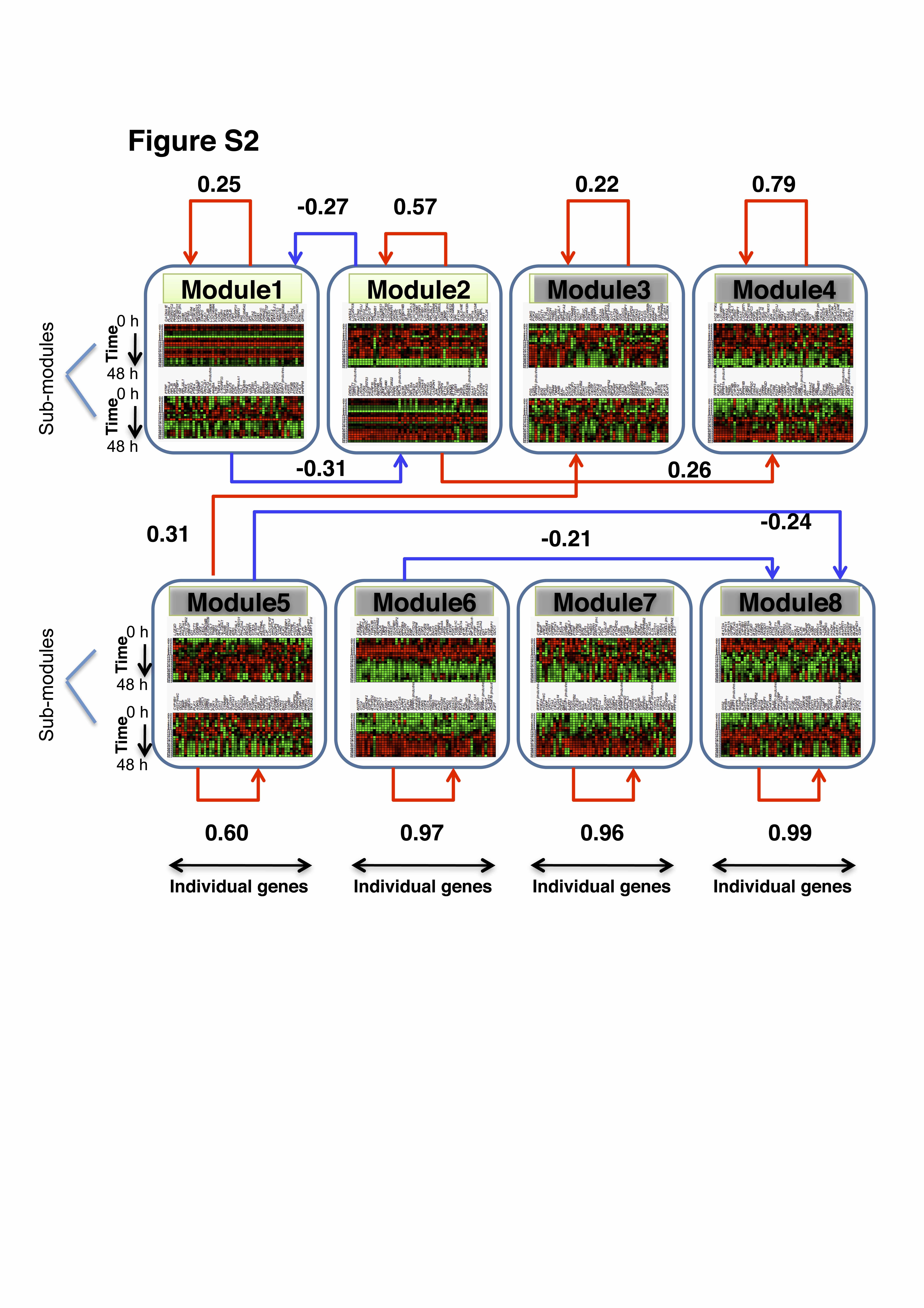

Supplement: Figure S2 — The eight module pairs in the EGF-signaling SSM. The time course changes in the expression levels of the 1,500 genes are classified into 8 expression patterns, called modules, that include a group of genes showing similar expression patterns. The most representative 100 genes for each module are shown. The expression pattern of each gene is vertically arranged. Each module is composed of two pairs of sub-modules that contain mirrored images of time-course gene expression patterns. Green indicates low expression compared to the average expression of each gene, and red indicates high expression compared to the average. Based on the assumption that genes belonging to the same module are under similar regulatory mechanisms, genes in a module regulate genes in every other module at each time point by the estimated regulation coefficients that are defined for each module (the estimated regulation coefficients are indicated as numbers on the red and blue arrows indicating positive and negative regulations, respectively). (JPG) [file pone.0043923.s002.jpg]

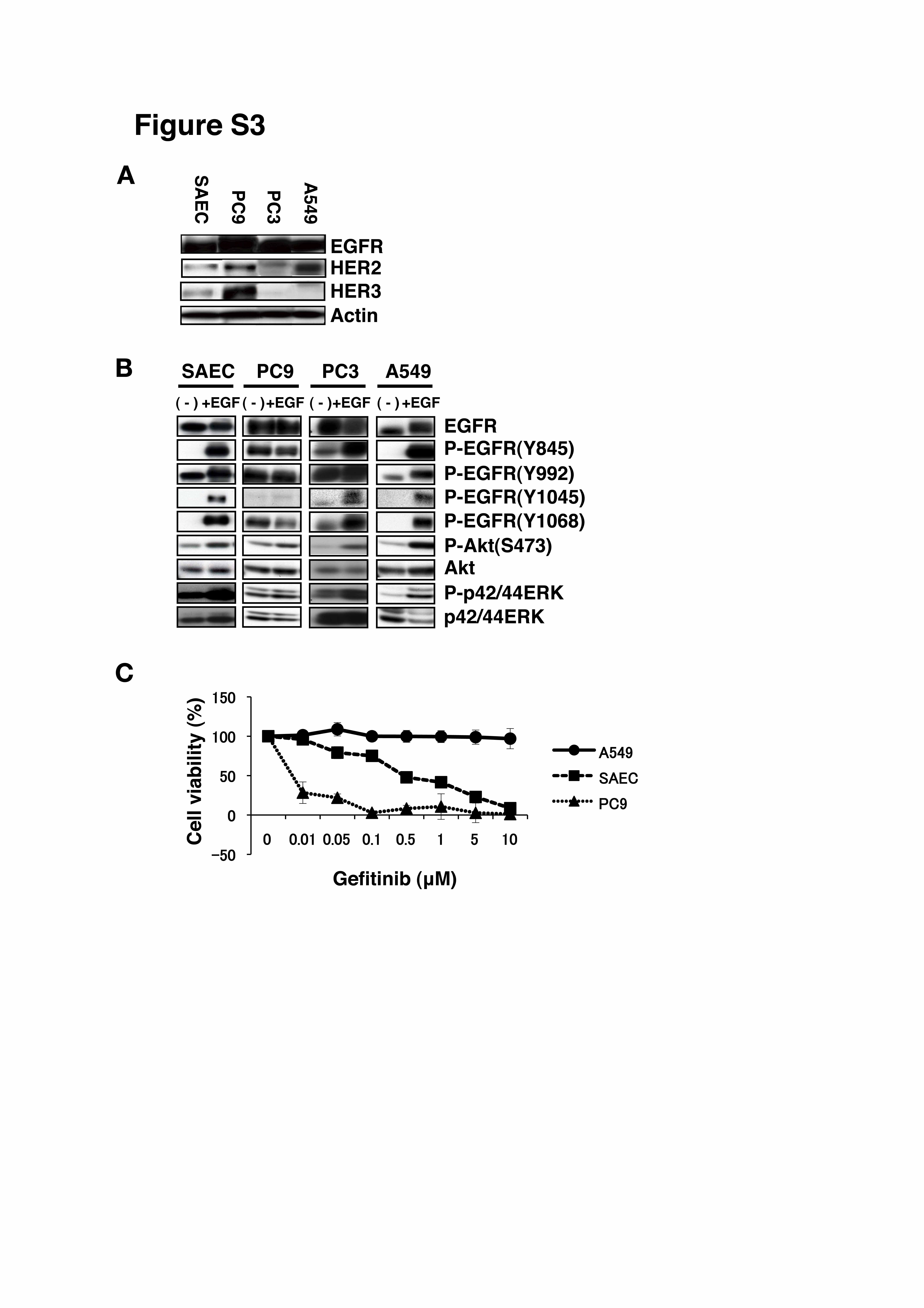

Supplement: Figure S3 — Profiles of human primary small airway epithelial cells (SAECs). (A) Expression levels of the epidermal growth factor receptor (EGFR) family members. Western blotting was performed using specific antibodies, as indicated on the right. (B) Phosphorylation of EGFR, Akt, and ERK, upon stimulation with EGF, in various lung cancer cell lines and SAEC. After starvation for 24 h at 37°C, the cells were stimulated with EGF (100 ng/mL) for 5 min at 37°C. Western blotting was performed using specific antibodies, as indicated on the right. “P” indicates “phosphorylated.” (C) Cell growth inhibition by gefitinib in a dose-dependent manner. Cell numbers were determined using a CellTiter 96® after incubation at 37°C for 72 h with a growth medium containing gefitinib. The results represent the means ± S.D. of several independent experiments. (JPG) [file pone.0043923.s003.jpg]

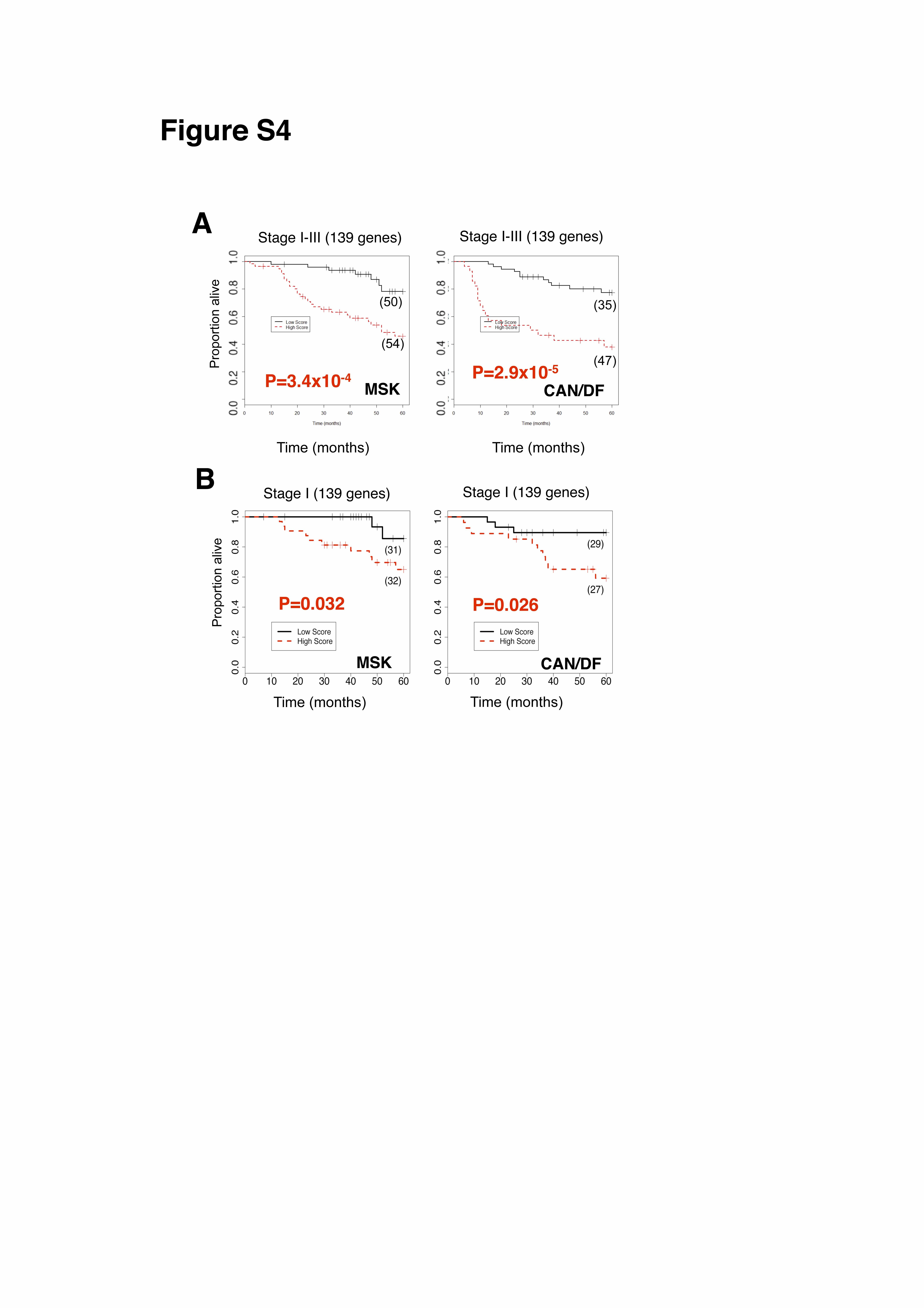

Supplement: Figure S4 — Kaplan-Meier plot survival estimates. (JPG) [file pone.0043923.s004.jpg]

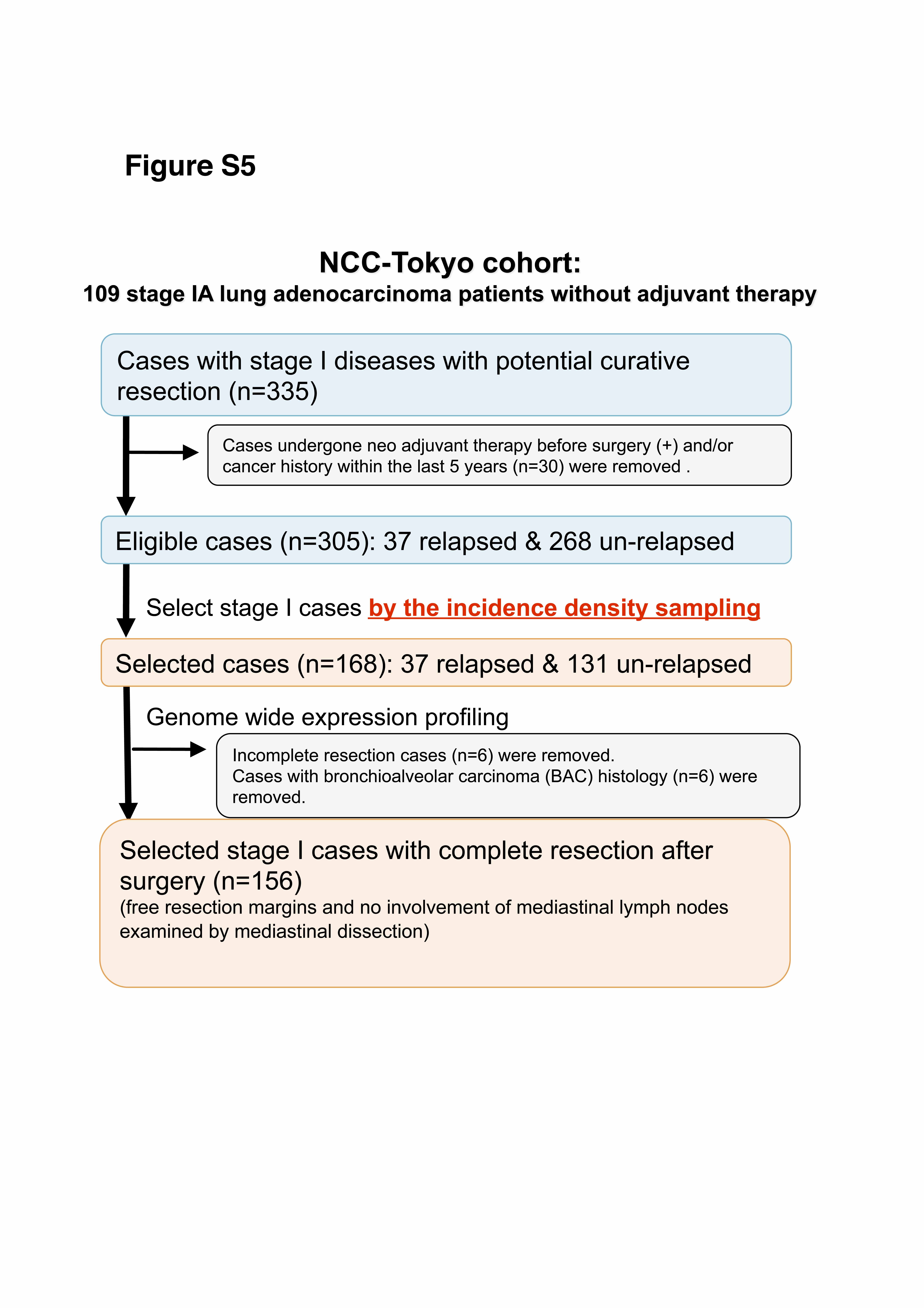

Supplement: Figure S5 — Selection of eligible cases of the NCC-Tokyo cohort consisting of 156 stage I lung adenocarcinoma patients without BAC histology and adjuvant therapy (22). (JPG) [file pone.0043923.s005.jpg]

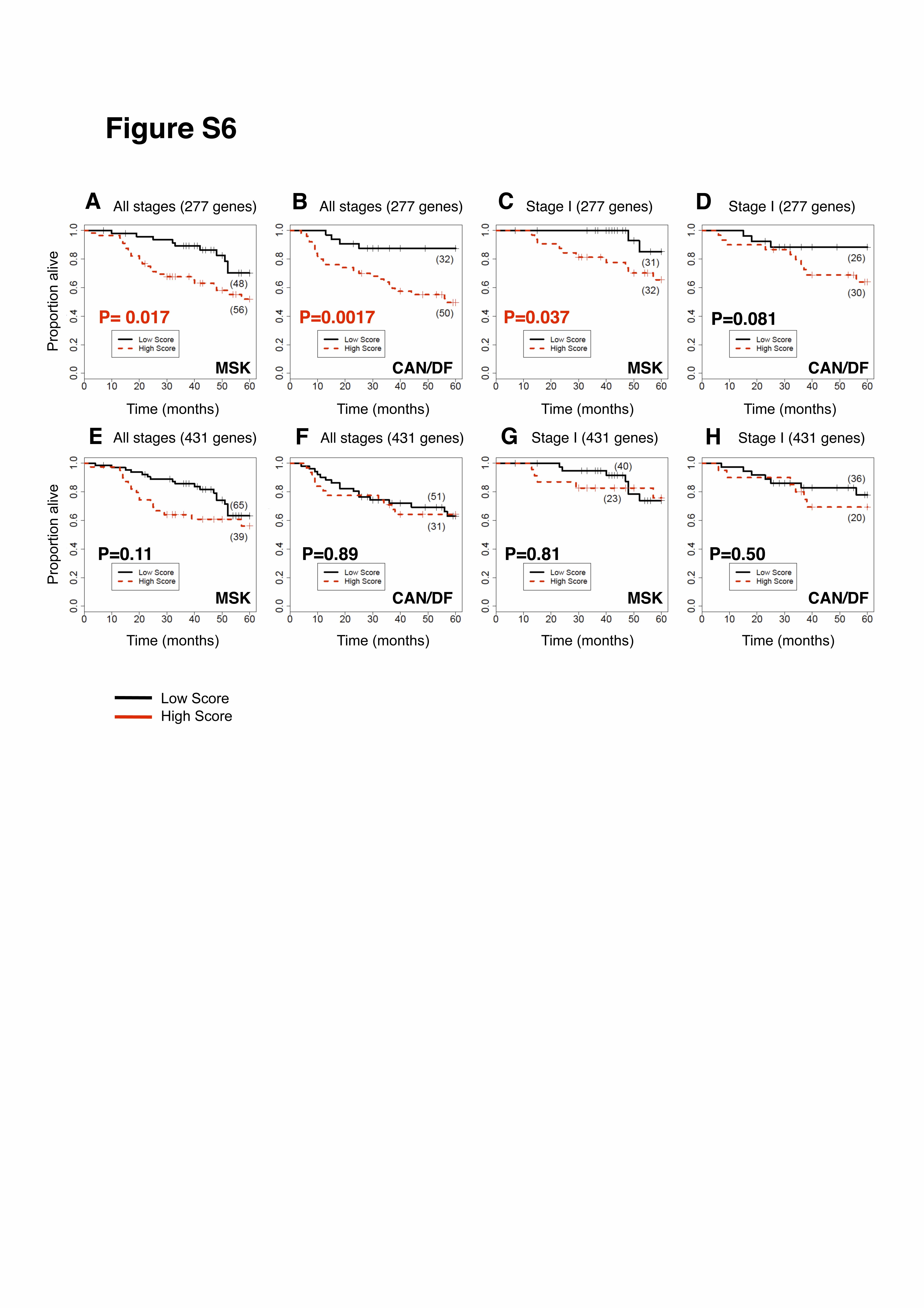

Supplement: Figure S6 — Comparison of the risk scoring model based on the gefitinib-sensitive genes and gefitinib-insensitive genes. A risk scoring model based on the 431 geftinib-insensitive gene signature was constructed. The prognostic ability of the two models: the 277 gefitinib-sensitive gene signature and 431 gefitinib-insensitive gene signature by using the two validation test sets are presented. The 277-gene signature was useful for predicting the survival of patients at all stages in both validation data sets and for the stage I MSK data set (P<0.05, as indicated in red in Figure S6A–C), except for the stage I CAN/DF data set (P = 0.081 in Figure S6D). Conversely, it was not possible to predict the survival for any stage at all when the 431 gefitinib-insensitive gene signature was used (high P-values, P>0.1 in Figure S6E–H). (JPG) [file pone.0043923.s006.jpg]
