# Supplementary material for: Epidermal Growth Factor Receptor Tyrosine Kinase Defines Critical Prognostic Genes of Stage I Lung Adenocarcinoma
Source: PLoS One. 2012 Sep 19;7(9):e43923. doi: 10.1371/journal.pone.0043923 (PMC3446964; doi:10.1371/journal.pone.0043923)
Supplement: Information S1 — Supporting information. (DOC) [file pone.0043923.s014.doc]

**Supporting information:**

**Materials and methods**

**Cell culture, growth assay and western blotting**

Recombinant human epidermal growth factor (EGF) was purchased from Millipore (Billerica, MA, USA). Gefitinib was extracted from Iressa® tablets (AstraZeneca, London, UK). Normal human small airway epithelial cells (SAECs) were purchased from Lonza Walkersville (Walkersville, MD, USA) and grown in SAGM medium (Cambrex, East Rutherford, NJ, USA). Lung cancer cells were grown in RPMI1640 medium (Nacalai Tesque, Kyoto, Japan) supplemented with 10% fetal bovine serum (FBS; JRH Bioscience, Lenexa, KS, USA), 100 U/mL penicillin (Nacalai Tesque) and 100 μg/mL streptomycin (Nacalai Tesque). Cells were seeded on 96-well plates with a growth medium containing the materials to be tested followed by incubation at 37 °C for 24 or 72 h. Cell numbers were determined using a CellTiter 96® (Promega, Madison, WI, USA). For experiments using SAECs, western blotting was performed as described [supplementary ref. (1)] with a specific antibody.

**Antibodies**

Anti-human EGF receptor (EGFR) Ab was purchased from Medical & Biological Laboratories (Nagoya, Japan). Anti-c-ErbB2/c-Neu (Ab-3) Ab was purchased from Calbiochem (Darmstadt, Germany). Anti-erbB-3/HER-3 (clone 2F12) Ab was purchased from Upstate Biotechnology (Lake Placid, NY, USA). Anti-phospho-p44/42 mitogen-activated protein kinase (MAPK) (Thr202/Tyr204) Ab, anti-p44/42 ERK Ab, anti-phospho-Akt (Ser473) Ab, anti-Akt Ab, anti-phospho-EGFR (Tyr845) Ab, anti-phospho-EGFR (Tyr992) Ab, anti-phospho-EGFR (Tyr1045) Ab, anti-phospho-EGFR (Tyr1068) Ab, anti-phospho-HER2/ErbB2 (Tyr1248) Ab, anti-phospho-HER3/ErbB3 (Tyr1289) (21D3) Ab, and anti-phospho-Stat3 (Tyr705) (D3A7) Ab were purchased from Cell Signaling Technology (Danvers, MA, USA). Horseradish peroxidase (HRP)-conjugated anti-mouse/rat/rabbit immunoglobulin G (IgG) Ab (Amersham Biosciences, Piscataway, NJ, USA) and HRP-conjugated anti-goat IgG Ab (Santa Cruz Biotechnology, Santa Cruz, CA, USA) were used as secondary antibodies.

**Microarray analysis of SAECs**

Total RNA was isolated from each sample by TRIzol® reagent (Invitrogen, Carlsbad, CA, USA) according to the manufacturer’s directions. The quality of the total RNA was evaluated on a 2100 Bioanalyzer (Agilent Technologies, Santa Clara, CA, USA). RNA samples were labeled using the Agilent Low RNA Input Linear Amplification Kit (Agilent Technologies) according to the manufacturer’s directions. Briefly, amplification and labeling of total RNA (500 ng) was performed using Cyanine 3-CTP. Hybridization was carried out using the Gene Expression Hybridization Kit (Agilent Technologies), according to the manufacturer’s directions. Briefly, a cRNA sample (1.65 μg) was subjected to fragmentation (30 min at 60 °C) and then hybridization on a 44K Agilent Whole Human Genome Oligo Microarray (G41112F) (Agilent Technologies). RNA samples with an RNA integrity number (RIN) >8 were used for further analysis. Hybridization was carried out on a 44K Agilent Whole Human Genome Oligo Microarray (G41112F) (Agilent Technologies). Microarray data acquisition and processing are described in Supporting information.

**Microarray data acquisition and processing for experiments using SAECs**

Microarrays were scanned with a dynamic autofocus microarray scanner (Agilent DNA Microarray Scanner, Agilent Technologies) using the default parameters (Green PMT was set to 100% and the scan resolution was set to 5 μm). Feature Extraction Software v9.1 (Agilent Technologies) was used to obtain a raw signal value and a quality flag (Present [P], Marginal [M], or Absent [A]). Median shift normalization was applied to the signals from each microarray (the median of the processed signals on a microarray is set to one). Then, we applied a Box-Cox transformation, which is $y\left(\lambda\right)=\frac{y^{\lambda}-1}{\lambda}$ where y is a signal value and $\lambda$ is a transformation parameter, for each microarray to further normalize the data. The transformation parameter $\lambda$ was set to 0.25. All 41,000 probes were mapped to 19,267 genes. To construct one-to-one reliable correspondence between these 41,000 probes and 19,267 genes, we selected one probe for each gene that had the largest number of P flags. If more than two probes had the same number of P flags, the probe with the maximum mean value expression throughout all the experiments was selected. The levels of gene expression were evaluated by the quality flags at each point.

**Selection of 1,500 genes for State Space Model (SSM) analysis**

First, 579 genes were selected based on a criterion that showed a >1.5-fold difference in expression levels between EGF-treated and untreated cells at any time point during an early phase from 0 to 9 hours (**Figure S1**). Another 190 genes were selected based on the criterion that their involvement in EGF signaling has been described in >2 independent literature reports among a total of ~400 literature reports in the PubMed database (<http://www.ncbi.nlm.nih.gov/sites/entrez?db=pubmed>). Another 802 genes were selected based on a criterion that they showed a potential to interact with the above 579+190 genes/proteins in signal transduction pathways in the Ingenuity Pathways Analysis (IPA) software ([http://www.ingenuity.com](http://www.ingenuity.com/); Ingenuity Systems, Inc., Redwood City, CA, USA). Among the 190+802 genes, 597 genes that showed measurable levels of expression in EGF-treated cells at most of the time points were selected. Finally, additional 324 genes were selected based on a criterion that they were top genes by variance in EGF-treated cells (**Supporting information, Gene selection procedure**).

**Gene selection procedure**

IPA (Ingenuity Systems) is a software platform that draws molecular networks in which molecules encoded by the genes of interest are involved based on the molecule interaction information maintained in the Ingenuity Pathway Knowledge Base (IPKB) [supplementary refs. (2, 3)]. We used IPA with the default parameter settings. We found that the 579+190 molecules are involved in the 41 Ingenuity canonical pathway networks and found that the 579+190 molecules interact with the additional 802 molecules. We next examined expression levels of genes encoding the 190+802 molecules and selected 597 genes among them if the number of P flags (measurable expression levels) was more than 28 among the total 32 data points, which included gene expression data from 19 time points and several cell replications in EGF-treated cells.

We selected 324 genes with large variances in the EGF-treated data as follows. We selected genes among the 19,267 genes, total number of genes analyzed by DNA microarray, if the number of P flags was more than 28 in the total 32 data points in EGF-treated cells. As a result, 12,082 genes were selected. We calculated the variance value of each gene based on its time course expression data, and then selected additional 324 genes in order of variance from largest to smallest.

**State Space Model (SSM)**

*y_n_* is the *p*-dimensional vector of expression values of genes at time $n\in N_{\text{obs}}$ whose *i*th element, *y_i_*,*_n_,* represents the expression value of the *i*th gene at time *n*. In our case, *p* = 1500. Here $N_{\text{obs}}$ is the index set of the observed time points. To unravel the internal system in gene expressions, we fit the state space model to the time-course data by

$$\begin{matrix} y_{n}=Hx_{n}+w_{n}, n\in N_{\text{obs}}, \\ x_{n}=Fx_{n-1}+v_{n}, n\in N, \end{matrix}$$

where *H* and *F* are $p\times k$ and $k\times k$ matrices, respectively, *x_n_* is the *k* dimensional vector of state variables, and *w_n_* and *v_n_* are observation and system noises. The former equation is called the observation model and the latter is called the system model. The index set *N* $(N_{\text{obs}}\subset N)$ contains the system time points; the internal system of gene expressions follows the first-order Markov process on *N*. Note that we assume the number *k* is much smaller than the number of genes *p*. Each state variable in *x_n_*, therefore, can be considered as a model of a regulator’s activity; the dependencies between a regulator and genes are described by the matrix *H*. We should note that *x_n_* are not observed; therefore, we need to treat them as hidden variables. The state space models can map a number of genes into a smaller number of state variables by using the observation model and build a temporal dynamics model of gene expression profiles by the system model, simultaneously.

The model parameters, *H*, *F*, *R* (the covariance matrix for *w_n_*), *μ*_0_ and **_0_ (the mean and covariance matrix of the initial state variables *x*_0_) are estimated using an EM algorithm along with a Kalman filter and smoother [supplementary ref. (4)], but a problem remains for the choice of the dimension of the state vector, *k*. Some information criteria have been proposed for the choice of the dimension of the state vector. Unfortunately, two well known information criteria, Akaike’s information criterion (AIC) and Bayesian information criterion (BIC), could not determine the optimal dimension, because AIC and BIC monotonically decreased (smaller values of AIC or BIC indicate a better model) with respect to *k* and we could not find the minimum. We had technically replicated data at the first 11 time points in$N_{\text{obs}}$; therefore, we used one time course for training and tested the prediction power of the estimated SSM based on the other time-course data. Hence, we could find the optimal dimension of the state variable, $\hat{k}=8$, automatically and found 16 gene sets (positively or negatively related with the eight state variables) from 1,500 genes. We denote these gene sets by modules (**Figure S2**).

We can find the network between state variables, called the module network, based on the system model with the estimate $\hat{F}$. **Figure S2** represents the estimated module network in this EGF signaling gene regulation SSM; the edges (or arrows) represent temporal relations between modules. For example, the eighth module has three incoming edges from the outcoming fifth and sixth modules and itself, and the values next to the edges represent the estimated coefficients. We found that the estimated Markov process for the eighth module is *x*_8_,*_n_* = −0.24*x*_5_,*_n_*_−1_ –0.21*x*_6_,*_n_*_−1_ +0.99*x*_8_,*_n_*_−1_. Therefore, we found a possibility that the eighth module is repressed by the fifth and sixth modules and is capable of self-activation. The expression values of genes related to the eighth module will be partly generated by this system (since we did not perform model selection for mapping between gene and module, genes are related to all modules in varying degrees).

**SSM analysis**

To select the gene subsets, we used the data for SAECs that were treated with EGF ($Y^{\text{EGF}}=\left\{y_{i,n}^{\text{EGF}}\right\}$) or EGF and gefitinib (GFT; $Y^{\text{EGF-GFT}}=\left\{y_{i,n}^{\text{EGF-GFT}}\right\}$). These samples served as the control and the case, respectively. $y_{i,n}^{\text{EGF}}$ and $y_{i,n}^{\text{EGF-GFT}}$ represent the gene expression of the *i*th gene in $G_{1500}$ at time *n* (*i* = 1, …, 1,500; $n\in N_{\text{obs}}$; $N_{\text{obs}}$ is an index set for the observation time points with $\left|N_{\text{obs}}\right|=19$).

The selection procedure is as follows:

1. The EGF-response SSM is constructed by estimating parameters with $Y^{\text{EGF}}$.
2. $y_{i,n}^{\text{EGF}}$ and $y_{i,n}^{\text{EGF-GFT}}$ are predicted by the EGF-response SSM and prediction errors ($\varepsilon_{i,n}^{\text{EGF}}$ and $\varepsilon_{i,n}^{\text{EGF-GFT}}$, respectively) are obtained for each gene at each observation time point.
3. A pair of integrated *P*-values ($P_{\text{Int},i}^{\text{EGF}}$,$P_{\text{Int},i}^{\text{EGF-GFT}}$) for each gene is obtained by a significance test of the prediction errors. This test is based on a meta-analysis called Meta Gene Profiler [supplementary refs. (5, 6)] (http://metagp.ism.ac.jp/). $P_{\text{Int},i}^{\text{EGF}}$ represents the quality of the estimation for the EGF-response SSM. Meanwhile, $P_{\text{Int},i}^{\text{EGF-GFT}}$ represents the predictability of the time course-gene expression patterns of EGF and gefitinib-treated cells by using the EGF-response SSM.
4. By selecting a pair of integrated P-values ($P_{\text{Int},i}^{\text{EGF}}$,$P_{\text{Int},i}^{\text{EGF-GFT}}$) as a threshold, a subset of gefitinib-sensitive genes or gefitinib-insensitive genes is extracted.

**Survival analysis in detail**

**[Construction of classifiers with gene signatures]**

To construct the risk score function, we utilized a partial Cox regression (20) [supplementary ref. (7)] with a principal component analysis, in which gene expression values were converted by the risk score function to a risk score to shift a baseline hazard function in a Cox proportional hazards model. The Cox proportional hazards model was, $\lambda\left(t,X_{i}\right)=\lambda_{0}(t)\exp\left[f(X_{i})\right]$, where $\lambda\left(t,X_{i}\right)$ and $\lambda_{0}(t)$ are a hazard function for the *i*th patient and a common baseline hazard function, respectively, and where$f(X_{i})$ is the risk score function, where $X_{i}=\left[X_{i1},\cdots,X_{ip}\right]^{T}$ is a vector of gene expression values of the *i*th patient for a gene set including the *P* genes. If a risk score for the *i*th patient is $f(X_{i})>0$, the prognosis for the patient is considered worse. If a risk score for the *i*th patient is ≤0, the prognosis for the patient is considered better. Thus, those patients with a risk score $f(X_{i})>0$ were classified into a high-risk group, and those patients with a risk score $f(X_{i})\leq0$ were classified into a low-risk group in this study. In the partial Cox regression, the risk score function is specified as $f(X_{i})=\sum_{j=1}^{p} \beta_{j}^{*}\left(X_{ij}-\bar{x}_{j}\right)$, a linear combination of $X_{ij}$, where $\beta^{*}=\left[\beta_{1}^{*},\cdots,\beta_{p}^{*}\right]^{T}$ are weight coefficients and $\bar{x}_{j}=\frac{1}{N}\sum_{i=1}^{N} X_{ij}$is the mean expression of the *j*th gene in a patient group consisting of *N* patients. Given a set of data that includes the survival or censoring times and gene expression values for the patients, we can estimate the parameters $\beta^{*}$ by the partial Cox regression method, in which a single latent component was used for a partial least squares regression. We used one training set to construct the risk score function and several independent validation sets in this study. Thus, a risk score for the *i*th patient in a validation set is given by

$f\left(X_{i}^{\text{Valid}}\right)=\sum_{j=1}^{p} \beta_{j}^{\text{*Train}}\left(X_{ij}^{\text{Valid}}-\bar{x}_{j}^{\text{Train}}\right)$,

where $X_{i}^{\text{Valid}}=\left[X_{i1}^{\text{Valid}},\cdots,X_{ip}^{\text{Valid}}\right]^{T}$ are gene expression values of the patient in a validation set, and $\beta^{*\text{Train}}=\left[\beta_{1}^{*\text{Train}},\cdots,\beta_{p}^{*\text{Train}}\right]^{T}$ are weight parameters estimated by the partial Cox regression using the survival or censoring times and gene expression values for patients in the training set, and $\bar{x}_{j}^{\text{Train}}$ is a mean expression value of the *j*th gene among the training samples. Due to the difference in the array platform between the NCI and Duke studies, a mean expression value of the jth gene among the Duke set samples were used instead of $\bar{x}_{j}^{\text{Train}}$ for risk score calculation.

We used the combined data from University of Michigan (UM) and Moffitt Cancer Center (HSM) of the National Cancer Institute (NCI) data set as the training data set (18) [supplementary ref. (8)]. These gene expression values were measured by using the Affymetrix GeneChip microarray (HGU-133A, Affymetrix, Inc., Santa Clara, CA, USA) while our gene signatures were selected by using the gene expression data of SAECs analyzed by the Agilent microarray (G41112F, Agilent Technologies). After probe matching using the Entrez ID, many genes were found to be measured by multiple probes on the microarrays of the NCI data sets. For the partial Cox regression analysis, we used only one expression value measured by using a unique probe that shows the highest variance in expression levels in all the patients in the training data set. All of the survival analyses were carried out by using software R (http://www.R-project.org). For the partial Cox regression analysis, we used R scripts provided at the website [supplementary ref. (7)].

**[Construction of classifiers with clinical covariates]**

To obtain classifiers with clinical covariates (age, gender, stage) and gene expression signatures, we constructed a risk score function based on a Cox proportional hazard model. In this study, the clinical covariates (age, gender, stage) were treated as categorical data and indicated by dummy variables in a Cox proportional hazard model. The covariate age had four categories: (1) Age<49, (2) 49<Age<59, (3) 59<Age<69, (4) Age>69. The covariate gender had two categories: (1) Male, (2) Female. The covariate stage had four categories: (1) stage IA, (2) stage IB, (3) stage II, and (4) stage III. We also included the risk scores for a gene set (e.g., $f(X_{i}^{\text{Valid}})$) as a scalar covariate, which was calculated by the risk score function for the gene set. We made a risk score function by selecting a subset of the above covariates (e.g., [age, gender, stage], [age, gender, stage, gene set]).

The risk score for the *i*th patients in a validation set with an m-dimensional covariate vector was given by the following risk score function:

$g\left(Z_{i}^{\text{Valid}}\right)=\sum_{j=1}^{m} \hat{\beta}_{j}^{\text{Train}}Z_{ij}^{\text{Valid}}-\bar{g}^{\text{Train}}$,

where are weight parameters for the covariate vector in the Cox proportional hazard model estimated with a training data set consisting of K samples, and $\bar{g}^{\text{Train}}=K^{-1}\left(\sum_{k=1}^{K} \sum_{j=1}^{m} \hat{\beta}_{j}^{\text{Train}}Z_{kj}^{\text{Train}}\right)$ is the mean risk score of the training samples. We classified each patient in a validation set by risk score: the high-risk group with $g\left(Z_{i}^{\text{Valid}}\right)>0$ and the low-risk group with ≤0.

**Estimation of the area under the curve (AUC) with risk scores**

To evaluate predictive accuracies for overall survival by risk scores from the constructed risk score functions, we estimated the receiver operating characteristics (ROC) curve and the AUC for 3-year survival or relapse-free survival [supplementary ref. (9)]. AUC may have a value from 0.5 to 1.0. Larger AUC represents better prediction accuracy, i.e., higher discriminatory power. The calculations of ROC and AUC were carried out by using software R (http://www.R-project.org).

**Data preprocessing using the Duke dataset**

The gene expression values of this data set were generated using the Affymetrix microarray (U133Plus2.0, Affymetrix). The CEL file data are available at http://data.cgt.duke.edu/oncogene.php. All CEL files were imported into the software dChip and signal values for each chip were calculated by using the default values. Among 54,675 probes on each sample, 22,277 probes were extracted as common probes shared between the Duke data set and the NCI data set. Sixty-two control probes were further removed. Next, 22,215 (=22,277-62) signal values for each sample were quantile-normalized. We note that this data set was not used to fit the partial Cox regression model, and used only to predict the survival of the patients based on the fitted model using the NCI data set.

**NCC-Tokyo cohort**

A total of 168 stage I lung adenocarcinoma cases were selected from 335 stage I cases that underwent potential curative resection between 1998 and 2008 at the National Cancer Center Hospital (Tokyo, Japan) as follows (**Figure S5**). Among the 335 cases, 305 cases were eligible based on the criteria of not having been diagnosed with cancer in the 5 years before lung adenocarcinoma diagnosis and not having received any adjuvant therapies before relapse. The 305 stage I cases included 37 cases with relapse and 268 cases without relapse. To improve statistical efficiency and avoid bias, all 37 relapsed cases and 131 matched, unrelapsed cases were selected by the incidence density sampling method [supplementary ref. (10)]. They were subjected to expression profiling. Among them, 6 cases were excluded because incomplete resection was later found and 6 cases with BAC histology were further excluded. The rest of the 156 stage I cases had complete resection (i.e., free resection margins and no involvement of mediastinal lymph nodes examined by mediastinal dissection) and did not receive any adjuvant chemotherapy or radiotherapy before relapse. Details are described elsewhere (23) [supplementary ref. (11)]. One hundred nanograms of total RNA extracted from macro-dissected lung adenocarcinoma cells were analyzed by Affymetrix U133Plus2.0 arrays.

**Microarray analysis of lung adenocarcinoma tissues of the NCC-Tokyo cohort**

Lung adenocarcinoma tissues were snap-frozen after resection and stored in liquid nitrogen at the National Cancer Center Hospital (NCC). Total RNA was extracted from macro-dissected lung adenocarcinoma cells using TRIzol reagent (Invitrogen), purified by the RNeasy kit (Qiagen Inc., Valencia, CA, USA), and qualified with a 2100 Bioanalyzer (Agilent Technologies). All samples showed RNA Integrity Numbers >6.0 and were subjected to microarray experiments. One hundred nanograms of total RNA were labeled using Two-Cycle Target Labeling and Control Reagents (Applied Biosystems/Ambion, Austin, TX, USA) and analyzed by Affymetrix U133Plus2.0 arrays. This study was approved by the institutional review boards of the National Cancer Center and the Institute of Medical Science, University of Tokyo.

**Microarray data processing for lung adenocarcinoma tissues of the NCC-Tokyo cohort**

A two-step normalization was done by the previously described method (18) [supplementary ref. (8)]. Due to the difference of the measurement arrays, HGU-133A (22,215 probes) for the NCI data set and U133Plus2.0 (54,675 probes) for the NCC-Tokyo data set, CEL files of the latter set were summarized by the dChip algorithm [supplementary ref. (12)] and probes common to both types of arrays were selected. Next, the dChip-summarized expression values of the selected probes were quantile-normalized by using expression values of the array NCI_U133A_61L in the NCI set as a reference. The expression values of the reference array were normalized according to Shedden et al. (18) [supplementary ref. (8)]. This study was approved by the institutional review boards of the National Cancer Center and the Institute of Medical Science, University of Tokyo.

**Detection of mutations on *EGFR***

Genomic DNAs from lung adenocarcinoma tissues were analyzed for somatic mutations of exons 19 and 21 of the *EGFR* by the high-resolution melting (HRM) method as described [supplementary ref. (13)].

**IPA to identify overlapping pathways with the 139 genes**

In order to investigate pathways that significantly overlapped with the 139 genes we identified, we mapped those genes to the Ingenuity canonical pathway database using IPA software and identified significantly overlapped pathways (Benjamini-Hochberg false discovery rate-adjusted P-values with Fisher’s exact test ≤ 0.01). The list of identified pathways is included (**Table 4** and **Table S7**).

**Results**

**Profiles of human primary SAECs**

Expression levels of EGFR in SAECs were similar to those in lung cancer cell lines: PC9 (*EGFR* mutation), PC3 (*EGFR* wild-type) and A549 (*EGFR* wild-type) (**Figure S3A**). EGF-stimulated tyrosine phosphorylation on EGFR was observed in SAECs as seen in PC3 and A549, while tyrosine residues were constitutively phosphorylated in PC9 cells (**Figure S3B**). PC9 cells are more sensitive to gefitinib than A549 cells, as previously reported [supplementary refs. (14, 15)], while SAECs showed an intermediate sensitivity (**Figure S3C**). These results indicate that SAECs retain EGF signaling pathways.

**Comparison of the risk scoring model based on the gefitinib-sensitive genes and**

**gefitinib-insensitive genes**

As a control, we also constructed a risk scoring model based on the 431 geftinib-insensitive gene signature. Then we tested the prognostic ability of the two models: the 277 gefitinib-sensitive gene signature and 431 gefitinib-insensitive gene signature by using the two validation test sets. We found that the 277-gene signature was useful for predicting the survival of patients at all stages in both validation data sets and for the stage I MSK data set (P<0.05, as indicated in red in **Figure S6A-C**), except for the stage I CAN/DF data set (P=0.081 in **Figure S6D**). Conversely, it was not possible to predict the survival for any stage at all when we used the 431 gefitinib-insensitive gene signature (high P-values, P>0.1 in **Figure S6E-H**). Thus, the gefitinib-sensitive genes, but not the gefitinib-insensitive genes, include key genes for the prognosis of lung adenocarcinomas.

**References**

1. Gotoh N, Tojo A, Muroya K, et al. Epidermal growth factor-receptor mutant lacking the autophosphorylation sites induces phosphorylation of Shc protein and Shc-Grb2/ASH association and retains mitogenic activity. Proc Natl Acad Sci U S A 1994;91:167-71.

**2. Calvano SE, Xiao W, Richards DR, et al. A network-based analysis of systemic inflammation in humans. Nature 2005;437:1032-7.**

**3. Ficenec D, Osborne M, Pradines J, et al. Computational knowledge integration in biopharmaceutical research. Brief Bioinform 2003;4:260-78.**

**4. Yamaguchi R, Yoshida R, Imoto S, Higuchi T, Miyano S. Finding module-based gene networks with state-space models - mining high-dimensional and short time-course gene expression data. IEEE Signal Processing Magazine 2007;24:37-46.**

**5. Yamaguchi R, Yamamoto M, Imoto S, et al. Identification of activated transcription factors from microarray gene expression data of Kampo medicine-treated mice. Genome Inform 2007;18:119-29.**

**6. Gupta PK, Yoshida R, Imoto S, Yamaguchi R, Miyano S. Statistical absolute evaluation of gene ontology terms with gene expression data. Lecture Notes in Bioinformatics 2007;4463:146-57.**

**7. Li H, Gui J. Partial Cox regression analysis for high-dimensional microarray gene expression data. Bioinformatics 2004;20 Suppl 1:i208-15.**

**8. Shedden K, Taylor JM, Enkemann SA, et al. Gene expression-based survival prediction in lung adenocarcinoma: a multi-site, blinded validation study. Nat Med 2008;14:822-7.**

**9. Heagerty PJ, Lumley T, Pepe MS. Time-dependent ROC curves for censored survival data and a diagnostic marker. Biometrics 2000;56:337-44.**

**10. Lubin JH GM. Biased selection of controls for case-control analyses of cohort studies. . Biometrics 1984;40:63-75.**

**11. Okayama H, Khono T, Ishii Y, et al. Identification of genes up-regulated in ALK-positive and EGFR/KRAS/ALK-negative lung adenocarcinoma. Cancer Res in press.**

**12. Li C, Wong WH. Model-based analysis of oligonucleotide arrays: expression index computation and outlier detection. Proc Natl Acad Sci U S A 2001;98:31-6.**

**13. Takano T, Ohe Y, Tsuta K, et al. Epidermal growth factor receptor mutation detection using high-resolution melting analysis predicts outcomes in patients with advanced non small cell lung cancer treated with gefitinib. Clin Cancer Res 2007;13:5385-90.**

**14. Amann J, Kalyankrishna S, Massion PP, et al. Aberrant epidermal growth factor receptor signaling and enhanced sensitivity to EGFR inhibitors in lung cancer. Cancer Res 2005;65:226-35.**

**15. Koizumi F, Shimoyama T, Taguchi F, Saijo N, Nishio K. Establishment of a human non-small cell lung cancer cell line resistant to gefitinib. Int J Cancer 2005;116:36-44.**

**Supporting Information Legends**

**Figure S1**

Gene selection procedure.

**Figure S2**

The eight module pairs in the EGF-signaling SSM. The time course changes in the expression levels of the 1,500 genes are classified into 8 expression patterns, called modules, that include a group of genes showing similar expression patterns. The most representative 100 genes for each module are shown. The expression pattern of each gene is vertically arranged. Each module is composed of two pairs of sub-modules that contain mirrored images of time-course gene expression patterns. Green indicates low expression compared to the average expression of each gene, and red indicates high expression compared to the average.

Based on the assumption that genes belonging to the same module are under similar regulatory mechanisms, genes in a module regulate genes in every other module at each time point by the estimated regulation coefficients that are defined for each module (the estimated regulation coefficients are indicated as numbers on the red and blue arrows indicating positive and negative regulations, respectively).

**Figure S3**

Profiles of human primary small airway epithelial cells (SAECs). (A) Expression levels of the epidermal growth factor receptor (EGFR) family members. Western blotting was performed using specific antibodies, as indicated on the right. (B) Phosphorylation of EGFR, Akt, and ERK, upon stimulation with EGF, in various lung cancer cell lines and SAEC. After starvation for 24 h at 37°C, the cells were stimulated with EGF (100 ng/mL) for 5 min at 37°C. Western blotting was performed using specific antibodies, as indicated on the right. “P” indicates “phosphorylated.” (C) Cell growth inhibition by gefitinib in a dose-dependent manner. Cell numbers were determined using a CellTiter 96® after incubation at 37°C for 72 h with a growth medium containing gefitinib. The results represent the means ± S.D. of several independent experiments.

**Figure S4**

Kaplan-Meier plot survival estimates.

**Figure S5**

Selection of eligible cases of the NCC-Tokyo cohort consisting of 156 stage I lung adenocarcinoma patients without BAC histology and adjuvant therapy (22).

**Figure S6**

Kaplan-Meier plot survival estimates.

**Table S1**

1,500 genes with integrated P-values calculated by using the SSM and the probe ID on the Affymetrix U133A.

**Table S2**

Clinicopathological characteristics of non-small cell lung cancer (NSCLC) subjected to prognosis analysis.

**Table S3**

Risk scores in the training data set for the 139 genes for survival prediction.

**Table S4**

Risk scores in the MSK test set of stage I disease for the 139 genes for survival prediction.

**Table S5**

Risk scores in the CAN/DF test set of stage I disease for the 139 genes for survival prediction.

**Table S6**

Risk scores in National Cancer Center Hospital test set of stage I disease for the 139 genes for survival prediction.
